# CD146 expression is associated with a poor prognosis in human breast tumors and with enhanced motility in breast cancer cell lines

**DOI:** 10.1186/bcr2215

**Published:** 2009-01-05

**Authors:** Gwladys Zabouo, Anne-Marie Imbert, Jocelyne Jacquemier, Pascal Finetti, Thomas Moreau, Benjamin Esterni, Daniel Birnbaum, François Bertucci, Christian Chabannon

**Affiliations:** 1 Institut Paoli-Calmettes, Centre de Ressources Biologiques en Oncologie, Centre de Thérapie Cellulaire et Génique, Marseille 13009, France; 2 Inserm U891, Centre de Recherche en Cancérologie de Marseille, Equipe Recherche Clinique, Marseille 13009, France; 3 Institut Paoli-Calmettes, Département de Bio-pathologie, Marseille 13009, France; 4 Inserm U891, Centre de Recherche en Cancérologie de Marseille, Equipe Oncologie Moléculaire, Marseille 13009, France; 5 Institut Paoli-Calmettes, Département de Médecine, Marseille 13009, France; 6 Université de la Méditerranée, Marseille 13007, France; 7 Inserm CIC-B510, Centre d'Investigations Cliniques en Biothérapie, Marseille 13009, France

## Abstract

**Introduction:**

Metastasis is a complex process involving loss of adhesion, migration, invasion and proliferation of cancer cells. Cell adhesion molecules play a pivotal role in this phenomenon by regulating cell–cell and cell–matrix interactions. CD146 (MCAM) is associated with an advanced tumor stage in melanoma, prostate cancer and ovarian cancer. Studies of CD146 expression and function in breast cancer remain scarce except for a report concluding that CD146 could act as a tumor suppressor in breast carcinogenesis.

**Methods:**

To resolve these apparent discrepancies in the role of CD146 in tumor cells, we looked at the association of CD146 expression with histoclinical features in human primary breast cancers using DNA and tissue microarrays. By flow cytometry, we characterized CD146 expression on different breast cancer cell lines. Using siRNA or shRNA technology, we studied functional consequences of CD146 downmodulation of MDA-MB-231 cells in migration assays. Wild-type, mock-transfected and downmodulated transfected cells were profiled using whole-genome DNA microarrays to identify genes whose expression was modified by CD146 downregulation.

**Results:**

Microarray studies revealed the association of higher levels of CD146 with histoclinical features that belong to the basal cluster of human tumors. Expression of CD146 protein on epithelial cells was detected in a small subset of cancers with histoclinical features of basal tumors. CD146^+ ^cell lines displayed a mesenchymal phenotype. Downmodulation of CD146 expression in the MDA-MB-231 cell line resulted in downmodulation of vimentin, as well as of a set of genes that include both genes associated with a poor prognosis in a variety of cancers and genes known to promote cell motility. *In vitro *functional assays revealed decreased migration abilities associated with decreased CD146 expression.

**Conclusions:**

In addition to its expression in the vascular compartment, CD146 is expressed on a subset of epithelial cells in malignant breast. CD146 may directly or indirectly contribute to tumor aggressiveness by promoting malignant cell motility. Changes in molecular signatures following downmodulation of CD146 expression suggest that CD146 downmodulation is associated with the reversal of several biological characteristics associated with epithelial to mesenchymal transition, and the phenomenon associated with the metastatic process.

## Introduction

Metastasis is a complex process involving loss of adhesion, migration, invasion and proliferation of cancer cells that receive signals and interact with the extracellular matrix, neighboring cells and growth factors. Cell adhesion molecules play a pivotal role in metastasis by regulating cell–cell and cell–matrix interactions [1].

CD146 (or MCAM, Mel-CAM, MUC18, S-endo1) was first described on malignant melanomas [2]. CD146 is a 113 kDA membrane glycoprotein that belongs to the immunoglobulin superfamily. It contains five immunoglobulin-like domains, one transmembrane region and a short cytoplasmic tail. The presence of several protein kinase recognition motifs in the cytoplasmic domain suggests the involvement of CD146 in cell signaling [3]. CD146 mediates homotypic and heterotypic adhesion between cells, although the ligand or the counter receptor is not known [4]. CD146 is a component of the inter-endothelial junction [5], and is now recognized as a marker of mesenchymal cells [6]. Its role in endothelial development is suggested by studies in the zebra fish [7]. The direct or indirect role of CD146 in cell migration has been suggested by several observations [8]. A recent report supports the importance of CD146 as a marker of bone marrow stromal cells with the ability to transfer the hematopoietic microenvironment to heterotopic sites [9]. Finally, CD146 is expressed on a small subset of activated T cells [10].

CD146 is structurally related to gicerin, a molecule that promotes metastasis of lymphoma cells in chicken [11] and metastasis of mouse mammary carcinoma cells [12]. Forced expression of CD146 in nonmetastatic melanoma cell lines increases their metastatic ability in mouse models [13]. More recent reports indicate that CD146 is overexpressed on prostate cancer cells [14], and that CD146 overexpression increases metastasis of prostate cancer cells in nude mice [15]. CD146 is associated with advanced tumor stage in ovarian cancers and could be a poor-prognosis factor that predicts early tumor relapse [16]. In pulmonary adenocarcinomas, CD146 expression is associated with shorter patient survival [17].

Antibodies against CD146 inhibit tumor growth of different xenografted tumor models: melanoma [18] and leiomyosarcoma, pancreatic cancer or hepatocarcinoma [19]. More recently, vaccination against murine melanoma cells expressing CD146 was shown to protect mice from lethal doses of melanoma cells [20].

Studies of CD146 expression and function in breast cancer – the leading cause of cancer morbidity and mortality among women – remain scarce, and mostly focus on circulating endothelial cells [21] or on tumor neoangiogenesis [22]. A previous report demonstrated that CD146 is expressed on epithelial and myoepithelial cells, and on 100% of benign proliferative epithelial lesions of the breast, but in only 18% of breast carcinomas, leading to the conclusion that CD146 could act as a tumor suppressor in breast carcinogenesis [23].

To resolve these apparent discrepancies in the role of CD146 in various models of malignancies, we further investigated CD146 expression in malignant human breast tissues to determine whether CD146 was associated with any particular tumor subtype, or biological or clinical feature. Our data suggest a role for CD146 in cell motility and progression in breast cancers, consistent with its role in other malignancies.

## Materials and methods

### Patients

Breast tumor specimens were obtained from consecutive cancer patients treated at our institution, following informed consent and a review of the protocol by the Institut Paoli-Calmettes Comité d'Orientation Stratégique (Institutional Review Board). Histological types included ductal carcinomas, lobular, mixed, tubular, medullar and other types. The median age of patients was 59 years (range 24 to 94 years). Women were treated according to guidelines used in our institution: after surgery, 93% received locoregional radiotherapy, 51% received adjuvant chemotherapy (anthracyclin-based regimen in most cases) and 52% received adjuvant hormonotherapy (tamoxifen, most cases). Tumor tissues were obtained before the initiation of systemic therapy.

### Immunohistochemistry on breast cancer tissue microarrays

Tissue microarrays were prepared as described previously [24], and were evaluated by the mean score of a minimum two core biopsies for each case. Slides were evaluated under a light microscope by two independent observers on the Spot Browser device (Alphelys, Plaisir, France) and were rated by the quick score [25], except for the tyrosine kinase receptor ERBB2 status (HercepTest kit; Dako France S.A.S., Trappes, France). Internal positive controls such as epidermis or benign breast lobules were used. Estrogen receptor (ER) and progesterone receptor (PR) were considered positive when at least 1% of tumor cell nuclei were stained. ERBB2 staining was considered positive when limited to a membrane staining of more than 10% of tumor cells (scored as 1+, 2+ or 3+ according to intensity). Protein overexpression was considered for scores of 2+ and 3+. Epidermal growth factor receptor was scored positive if any membranous invasive carcinoma cell staining was observed. See Additional data file 1 for the mAbs used in the present study.

### Cell lines

Ten breast tumor cell lines were used in this study: BT-549, Hs578T, MCF-7, MDA-MB-231, MDA-MB-436, MDA-MB-453, T-47D, ZR-75-30 (all from American Type Culture Collection, Manassas, VA, USA), BrCa-MZ-02 [26] and SUM159PT (Asterand, Detroit, MI, USA).

ZR-75-30, T47D, BrCA-MZ-02, MDA-MB-453, MDA-MB-231, MDA-MB-436 and BT549 cells were cultured in RPMI (Cambrex, Verviers, Belgium) supplemented with 10% heat-inactivated FCS (Invitrogen, Paisley, UK). MCF-7 cells were cultured in the same medium supplemented with insulin (30 μg/ml; Sigma-Aldrich, St Louis, MO, USA). Sum159PT cells were cultured in the same medium (RPMI, FCS and insulin) supplemented with hydrocortisone (1 μg/ml). Hs578T cells were cultured in RPMI supplemented with 10% FCS, insulin (30 μg/ml) and glucose (2.5 g/l). All of the culture media contained 100 U/ml penicillin and 100 μg/ml streptomycin (Invitrogen).

The HBMEC cell line, a kind gift from B. Weksler (New York, USA), is derived from adult human bone marrow endothelial cells, following SV-40 immortalization, and was cultured as previously described [27,28].

### Flow cytometry

Analyses were conducted with a LSRII Flow cytometer (Becton-Dickinson Immunocytometry Systems, San Jose, CA, USA). Cells were incubated with mAbs (see Additional data file 1) for 30 minutes on ice, and with a phycoerythrin-labeled goat anti-mouse antibody (Beckman-Coulter, Miami, FL, USA) in case of unconjugated mAbs. Isotype controls were used to exclude false positive cells. Dead cells were gated out by staining with Dapi (1 μg/ml; Invitrogen).

### Quantitative RT-PCR

Total RNA was isolated from 1 × 10^6 ^to 2 × 10^6 ^cells using an RNA extraction kit (Macherey-Nagel GmBH & Co, Düren, Germany), denatured at 65°C for 10 minutes and reverse-transcribed using Superscript II reverse transcriptase (Invitrogen). Quantitative RT-PCR was carried out using the LightCycler 2.0 instrument and software version 4.0 (Roche Diagnostics, Meylan, France). The 20 μl reaction mixture contained 4 μl of 5× Master Mix (Roche Diagnostics), 0.5 μM each primer and 1 μl cDNA sample. After initial incubation at 95°C for 10 minutes, 60 cycles were carried out (10 s at 95°C, 10 s at 60°C, and 20 s at 72°C). To confirm differential expression observed with DNA microarray, the 21 downregulated genes were quantified by RT-PCR. The primers are listed in Additional data file 2.

In addition, the expression of 180 adhesion, migration and cytoskeleton genes (see Additional data file 3) was tested from 1 μg cDNA using SYBR Green reagent on an ABI7700 system (Applied Biosystems, Foster City, CA, USA). Specific primers were designed using the Primer Express Software (Applied Biosystems) and were spotted in 96-well plates, which were made available to us through a collaboration with Inserm U 576-Nice Régulations des réactions immunitaires et inflammatoires. Gene expression was normalized for RNA concentration with four endogenous genes (GAPDH, HPRT, ubiquitin, β-actin).

### RNA-interference mediated gene silencing

siRNA duplexes directed against CD146 [GenBank:NM_006500] were synthesized by Invitrogen (see Additional data file 2). Two negative controls were used: a mutated siRNA (si78mut) with a modification of four nucleotides, and a siRNA that recognizes the green fluorescent protein gene (siGFP).

Then 10^5 ^cells were plated in six-well culture dishes in 2.5 ml medium without antibiotics. Cells were transfected with a mixture of siRNA (10 nM) and Lipofectamine RNai/Max (Invitrogen) according to the manufacturer's protocol.

The 29mer shRNA expression vectors (see Additional data file 2) directed against CD146 were obtained from Origene Technologies, Inc. (Rockville, MD, USA). These vectors in pRS plasmid were amplified and purified with the Nucleobond PC 100 Kit (Macherey-Nagel). MDA-MB-231 cells were plated at 3 × 10^5 ^cells in six-well plates. Transfections were performed using Fugene-6 (Roche Diagnostics) as directed by the manufacturer. Forty-eight hours after transfection, puromycin (0.8 μg/ml; Sigma-Aldrich) was added. Transfected cell lines were grown in the presence of puromycin. Two negative control shRNA expression vectors were used in this study: the original vector plasmid [TR20003], and a vector containing a noneffective shRNA cassette against green fluorescent protein [TR30003].

### Cell migration assays

Before migration, cells were starved overnight in RPMI medium (Lonza, Walkersville, MD, USA). Migration was observed in transwell culture inserts of 6.5 mm diameter and 8 μm pore filters (Greiner Bio-One SAS, Courtaboeuf, France). Then 3 × 10^4 ^cells in 100 μl RPMI medium with 1% FCS (Invitrogen) were seeded in the upper compartment, and 600 μl RPMI 10% FCS were added to the lower chamber. Cells were allowed to migrate for 24 hours at 37°C. After removing cells on the upper side of the transwell, cells on the underside were stained with 0.1% crystal violet solution (Becton Dickinson) and were lysed with 10% acetic acid for quantification by densitometric measurement at 550 nm. In some experiments, cells were preincubated with an anti-CD146 mAb (S-Endo1; BioCytex, Marseilles, France) for 1 hour at 4°C and the mAb was present during the migration assay. Experiments were carried out in triplicate.

For transmigration assays, 40,000 HBMEC cells were established to confluence in 0.1% gelatin-coated transwells. Wild-type (30,000 cells) or genetically modified MDA-MB-231 cells in RPMI medium supplemented with 0.2% bovine serum albumin (Sigma-Aldrich) were seeded in the upper chamber. The lower chambers were filled with 600 μl RPMI 10% FCS. After 24 hours, staining was performed as for migration assays. In order to account for the possible migration of HBMEC cells, a blank well was included in all series.

For wound healing assays, 3.5 × 10^3 ^cells were seeded in 24-well plates and were grown to confluence. Cells were scrapped with a 200 μl micropipette tip (0 hours) and allowed to migrate for 24 hours. Each wound area was photographed (0 hours and 24 hours) using an Olympus IX70 inverted microscope equipped with a digital camera (Olympus France, Rungis, France). The percentage of the cell-free area was estimated with the use of ImageJ software [29].

### Gene expression profiling with DNA microarrays

Wild-type, mock-transfected and stably transfected cell lines were profiled using whole-genome DNA microarrays. Gene expression analyses were performed with Affymetrix U133 Plus 2.0 human oligonucleotide microarrays (Affymetrix, Santa Clara, CA, USA) containing over 47,000 transcripts and variants, including 38,500 well characterized human genes. Preparation of cDNA from 2 μg total RNA, hybridizations, washes and detection were carried out as recommended by the supplier. Scanning was performed with the Affymetrix GeneArray scanner, and quantification with Affymetrix GCOS software.

Expression data were analyzed by the Robust Multichip Average method in R using Bioconductor and associated packages [30]. A filtering process removed from the dataset the genes with low and poorly measured expression, retaining 18,041 probe sets. Supervised analysis compared expression profiles from the three control cell lines with those from the two experimental cell lines. In a first step, a Student's *t *test with false discovery rate correction retained probe sets differentially expressed between the two groups with a significance threshold of *P *< 0.05. In a second step, we measured for each significant probe set the fold change of mean expression levels between the control group and the experimental group. Results [GEO:GSE11951] were displayed simultaneously in a volcanoplot [31].

### Statistical analyses

Survival rates were estimated following the Kaplan–Meier method. Overall survival was calculated from the date of diagnosis until the date of death and was compared between groups with the log-rank test. Correlations between sample groups and histoclinical factors were calculated using the Fisher's exact test for qualitative variables with discrete categories. All statistical tests were two-sided at the 5% level of significance. Statistical analyses were performed using the survival package (version 2.30) in R software [32].

## Results

### CD146 is expressed in human primary breast tumors

**Figure 1:**
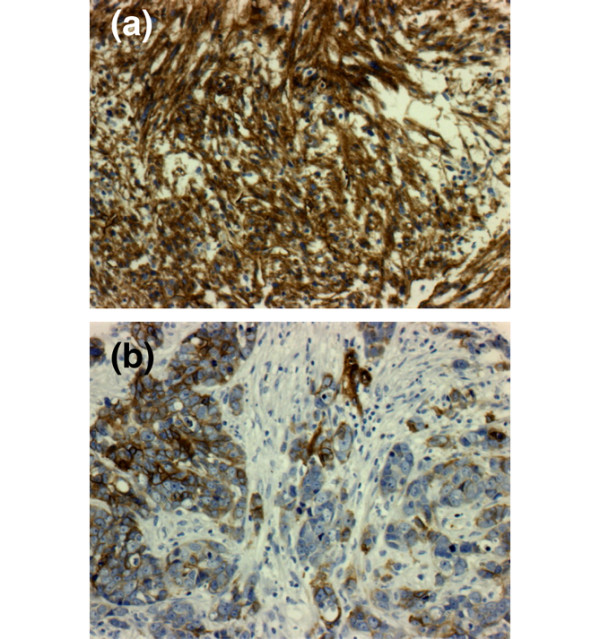
**CD146 protein expression in human primary breast cancer and specific survival**. Examples of CD146 staining for **(a) **a metaplastic carcinoma and **(b) **an invasive adenocarcinoma.

The study of 635 untreated primary breast tumors aggregated in two tissue microarrays revealed that 45 tumors (7%) were positive for CD146 staining in the epithelial compartment (Figure 1a,b). Most CD146-positive tumors were ductal carcinomas (78%). CD146 expression was strongly associated with high grade, with negativity for ER and PR, and with the triple-negative (ER^-^/PR^-^/ERBB2^-^) phenotype. Association with positivity for epithelial growth factor receptor, p53, P-cadherin and Moesin, together with negativity for GATA-3 and BCL2, indicated a pattern of basal tumors (Table 1) [33].

**Table 1:** Histoclinical correlations of breast cancers according to CD146 expression

Characteristic	DNA microarray		Tissue microarray	
		
	CD146-rich (n = 113)	*P *value*	CD146^+ ^(n = 45)	*P *value*
Age				
< 45 years	36/74	NS	9/85	NS
≥ 45 years	59/115		36/549	
Pathological type				
Ductal	92/179	NS	36/472	NS
Lobular	17/43		2/77	
Tubular			1/40	
Medullar			1/8	
Mixed			2/25	
Other			4/26	
Molecular subtype				
Basal	44/67	<0.0001		
ERBB2	15/31			
Luminal	32/93			
Normal	16/21			
Scarff Bloom and Richardson grade				
I + II	26/77	<0.0001	18/463	<0.0001
III	87/149		27/163	
Tumor size				
< 20	28/53	NS	17/281	NS
≥ 20	54/122		26/347	
Pathological axillary lymph node status				
Negative	52/90	NS		
Positive	54/122			
Immunohistochemistry estrogen receptor status				
Negative	72/119	<0.0001	28/141	<0.0001
Positive	41/108		13/460	
Immunohistochemistry progesterone receptor status				
Negative	83/137	<0.0001	35/208	<0.0001
Positive	30/90		9/362	
Immunohistochemistry ERBB2 status				
Negative	83/169	NS	40/515	NS
Positive	19/36		2/46	
Immunohistochemistry estrogen receptor/progesterone receptor/ERBB2 status				
Triple negative			25/76	<0.0001
Other			13/425	
Immunohistochemistry p53 status				
Negative			14/330	0.0046
Positive			15/124	
Immunohistochemistry Bcl2 status				
Negative			22/138	<0.0001
Positive			18/388	
Immunohistochemistry CD44 status				
Negative			1/136	0.0225
Positive			17/283	
Immunohistochemistry epithelial growth factor receptor status				
Negative			3/284	0.00011
Positive			9/81	
Immunohistochemistry GATA3 status				
Negative			30/203	0.00032
Positive			10/313	
Immunohistochemistry Moesin status				
Negative			4/339	<0.0001
Positive			13/65	
Immunohistochemistry P-cadherin status				
Negative			1/199	0.00021
Positive			14/173	

*To assess differences in clinicopathologic features between the two groups of patients, Fisher's exact test was used for qualitative variables with discrete categories.

**Figure 2:**
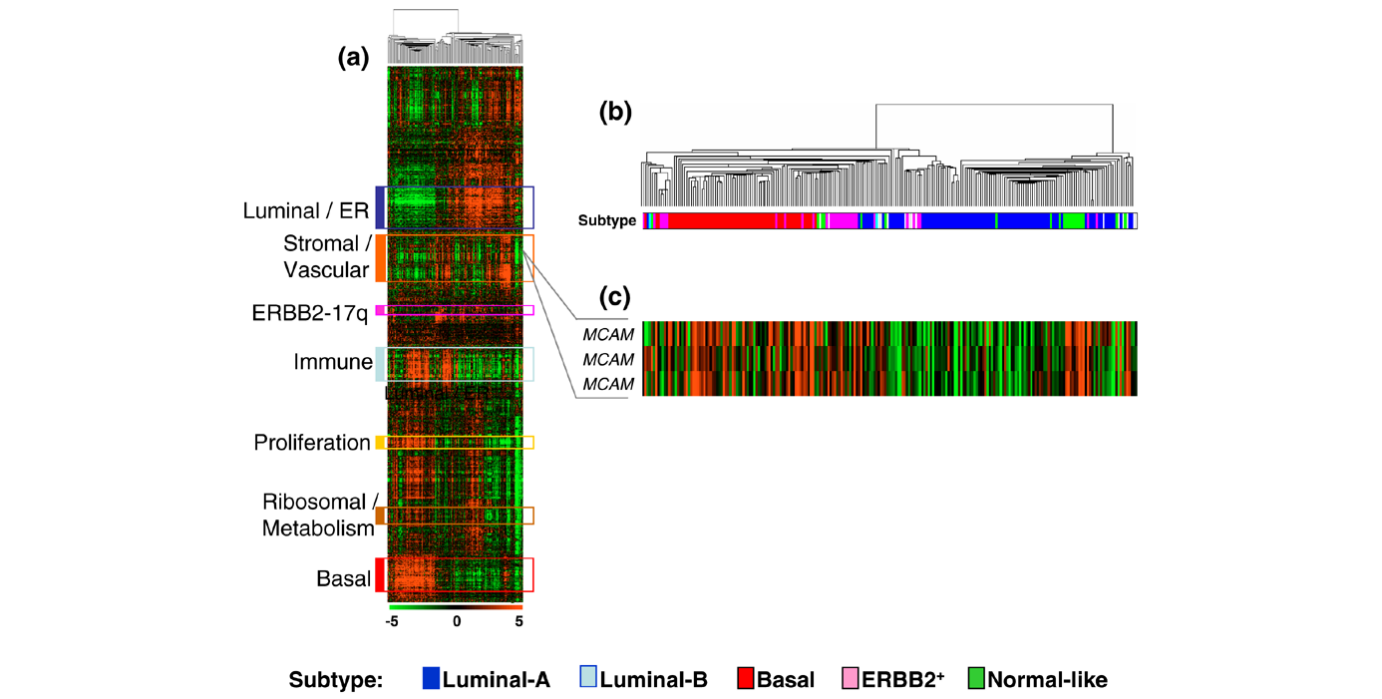
**CD146 mRNA expression in human primary breast tumors. (a) **Hierarchical clustering of 227 breast cancer tissue samples and 14,486 genes/Expressed Sequence Tags based on mRNA expression levels. Each row represents a gene, and each column represents a tumor. The expression level of each gene in a single tumor is relative to its median abundance across all tumors and is depicted according to a color scale shown at the bottom. Red and green, expression levels respectively above and below the median. The magnitude of deviation from the median is represented by the color saturation. The dendrogram of samples (above matrix) represents overall similarities in gene expression profiles and is magnified in (b), colored bars to the left, locations of seven gene clusters of interest. The stromal/vascular gene cluster (orange bar) includes MCAM. ER, estrogen receptor. **(b) **Dendrogram of breast cancer samples. **(c) **Expanded view of CD146/MCAM expression. Also see Additional data file 4.

Analysis of our previously published gene expression data of 227 breast cancer samples profiled using oligonucleotide microarrays [34] also supported this conclusion: an expanded view of the hierarchical clustering of our tumor series showed that CD146 is included in a stromal gene cluster enriched in mesenchymal and vascular genes (see Additional data file 4), and is overexpressed in basal tumors as compared with luminal tumors (Figure 2). With the median expression level of the corresponding probe set across all tumors as the cutoff point for defining a rich or poor tumor for CD146, CD146-rich tumors were more frequently grade III, ER-negative or PR-negative, and displayed a basal phenotype in 41% of cases versus 22% for CD146-poor tumors (Table 1).

**Figure 3:**
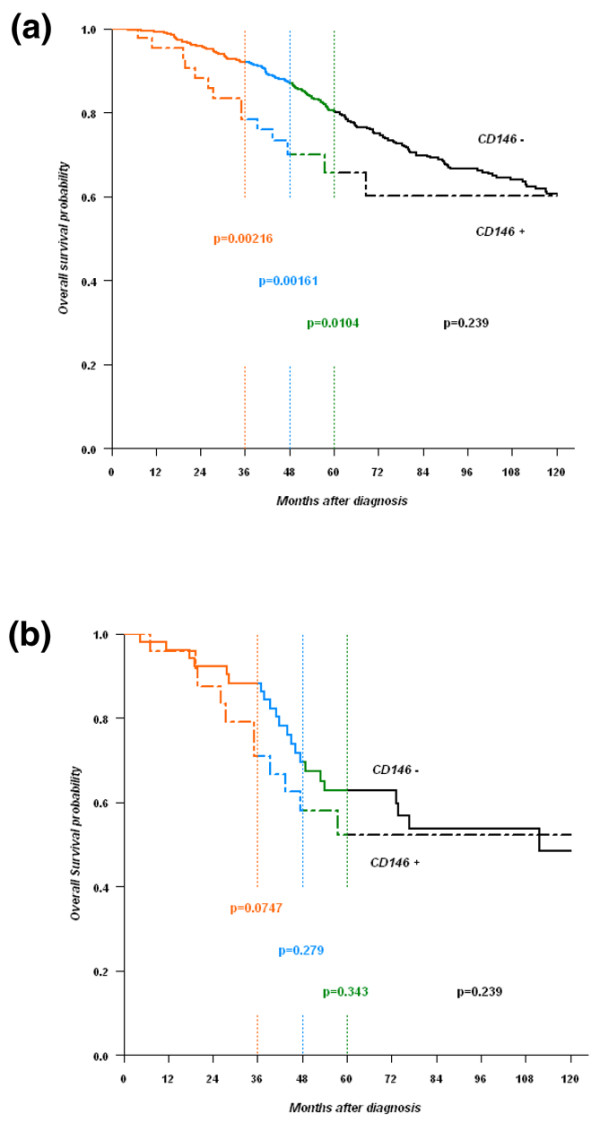
**Specific survival of patients with CD146^- ^and CD146^+ ^tumors**. CD146 expression was defined using immunohistochemistry on tissue microarrays. **(a) **All tumors. **(b) **Estrogen receptor-negative/progesterone receptor-negative/ERBB2-negative (triple-negative) tumors.

Kaplan–Meier analysis with a follow-up censored at 5 years indicated a statistically significant difference in the overall survival time between patients with positive and negative CD146 status, as assayed with immunohistochemistry on tissue microarrays (*P *= 0.0104, log-rank test; Figure 3a); after 5 years, survival rates no longer indicated a difference between the two subgroups. In triple-negative tumors there was a nonstatistically significant trend towards a shorter overall survival in CD146-positive tumors during the first 3 years (Figure 3b). An increased risk of death before 5 years is therefore associated with CD146 expression in the epithelial compartment of breast cancer tissues.

### CD146 is a marker of mesenchymal-like breast cancer cell lines

**Figure 4:**
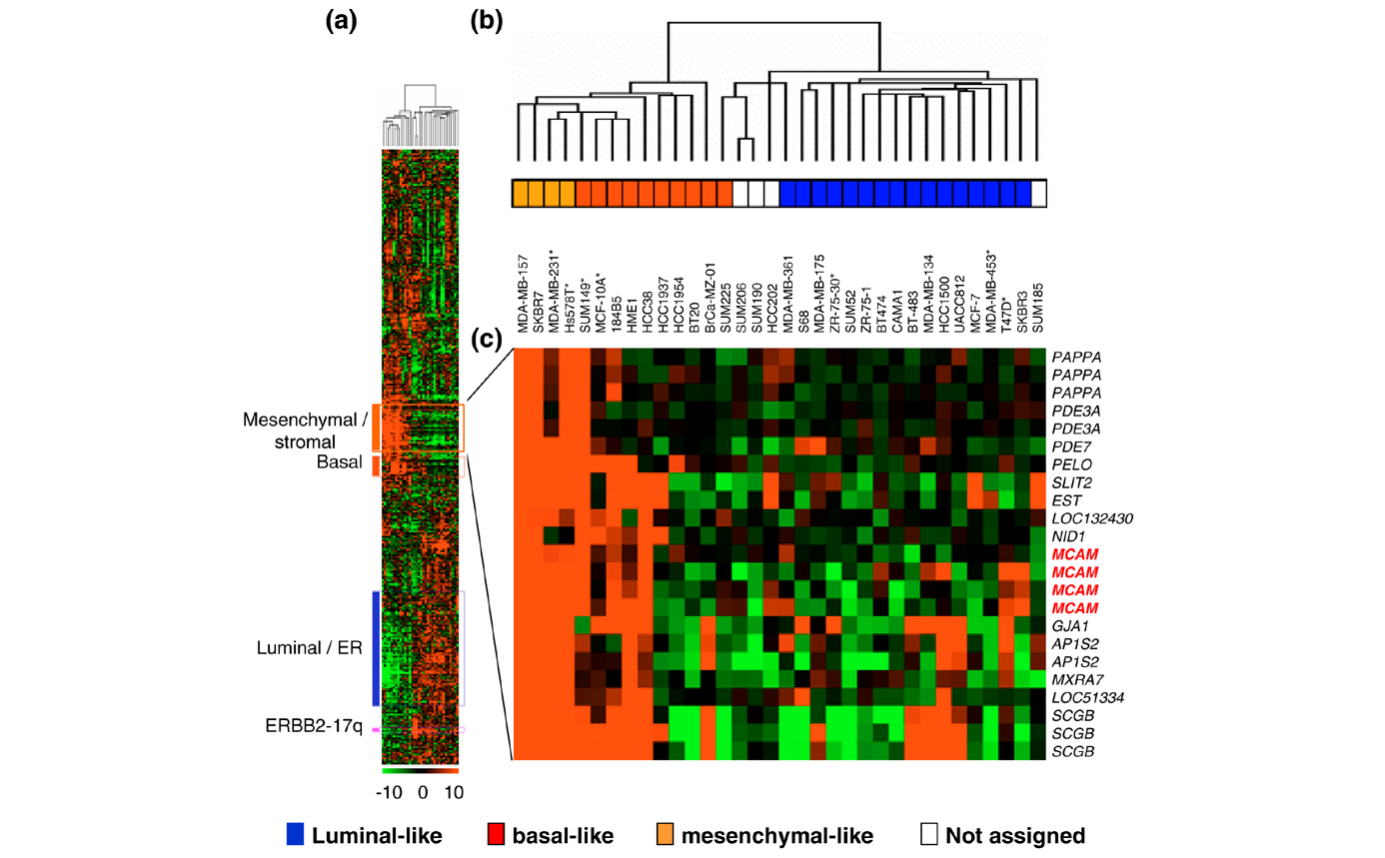
**CD146 mRNA expression in human breast cancer cell lines**. **(a) **Hierarchical clustering of 34 mammary cell lines and 13,976 genes/Expressed Sequence Tags based on mRNA expression levels. The legend is similar to Figure 2. Colored bars to the left, locations of four gene clusters of interest. The stromal/mesenchymal gene cluster (orange bar) includes MCAM. ER, estrogen receptor. **(b) **Dendrogram of cell lines. *Cell lines analyzed in the present study by flow cytometry for CD146 expression (low expression, MCF-7, ZR-75-30 and MDA-MB-453; high expression, MDA-MB-231, Hs578T and MCF-10A). **(c) **Expanded view of the stromal/mesenchymal gene cluster, which includes the four probe sets representing CD146/MCAM. Genes are referenced by their HUGO abbreviation as used in Entrez Gene.

DNA microarray analyses of 34 mammary cell lines [34] show the strong expression of CD146 in MDA-MB-231 and Hs578T cell lines, and its low expression in MCF-7, ZR-75-30 and MDA-MB-453 cells. An expanded view of the hierarchical clustering shows that CD146 is included in a stromal/mesenchymal gene cluster, near the basal gene cluster, and is overexpressed in mesenchymal-like cell lines as compared with other cell lines (Figure 4).

**Figure 5:**
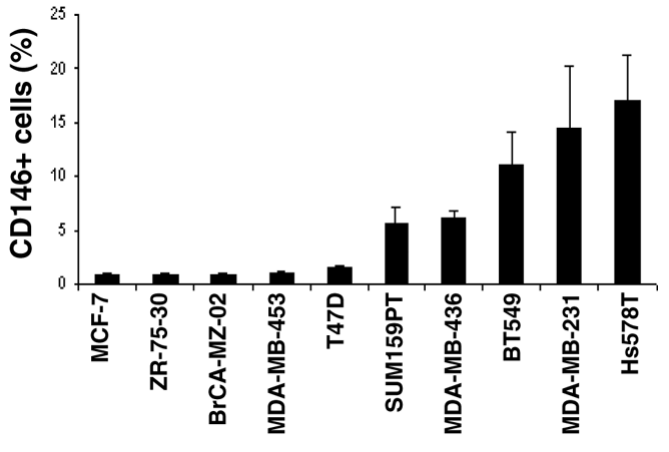
**CD146 expression in breast cancer cell lines**. Values indicate the specific mean fluorescence intensity (sMFI) ± standard error of the mean in at least six independent experiments, using the P1H12 mAb. The sMFI was defined as the ratio of the mean fluorescence intensity for the considered mAb over the mean fluorescence intensity obtained with the appropriate isotypic control.

We confirmed these results by flow cytometry analyses. Two subgroups of human mammary cancer cell lines were easily distinguishable on the basis of CD146 expression (Figure 5): MCF-7, ZR-75-30, BrCA-MZ-02, MDA-MB-453 and T47-D cell lines, which display epithelial characteristics, expressed CD146 at low levels (CD146^-^); and oppositely, MDA-MB-231, Hs578T, Sum159PT, MDA-MB-436 and BT-549 cell lines, which display mesenchymal characteristics, also expressed high levels of CD146 (CD146^+^). Of note, the T47D cell line was negative for CD146 protein expression while the mRNA was detected in microarray and RT-PCR analyses, thus suggesting the possibility of post-transductional regulation.

### Downmodulation of CD146 expression in the MDA-MB-231 mammary cancer cell line results in decreased migration

For further experiments, the MDA-MB-231 cell line was used as a prototypic mesenchymal and invasive cell line, spontaneously expressing high levels of CD146.

**Figure 6:**
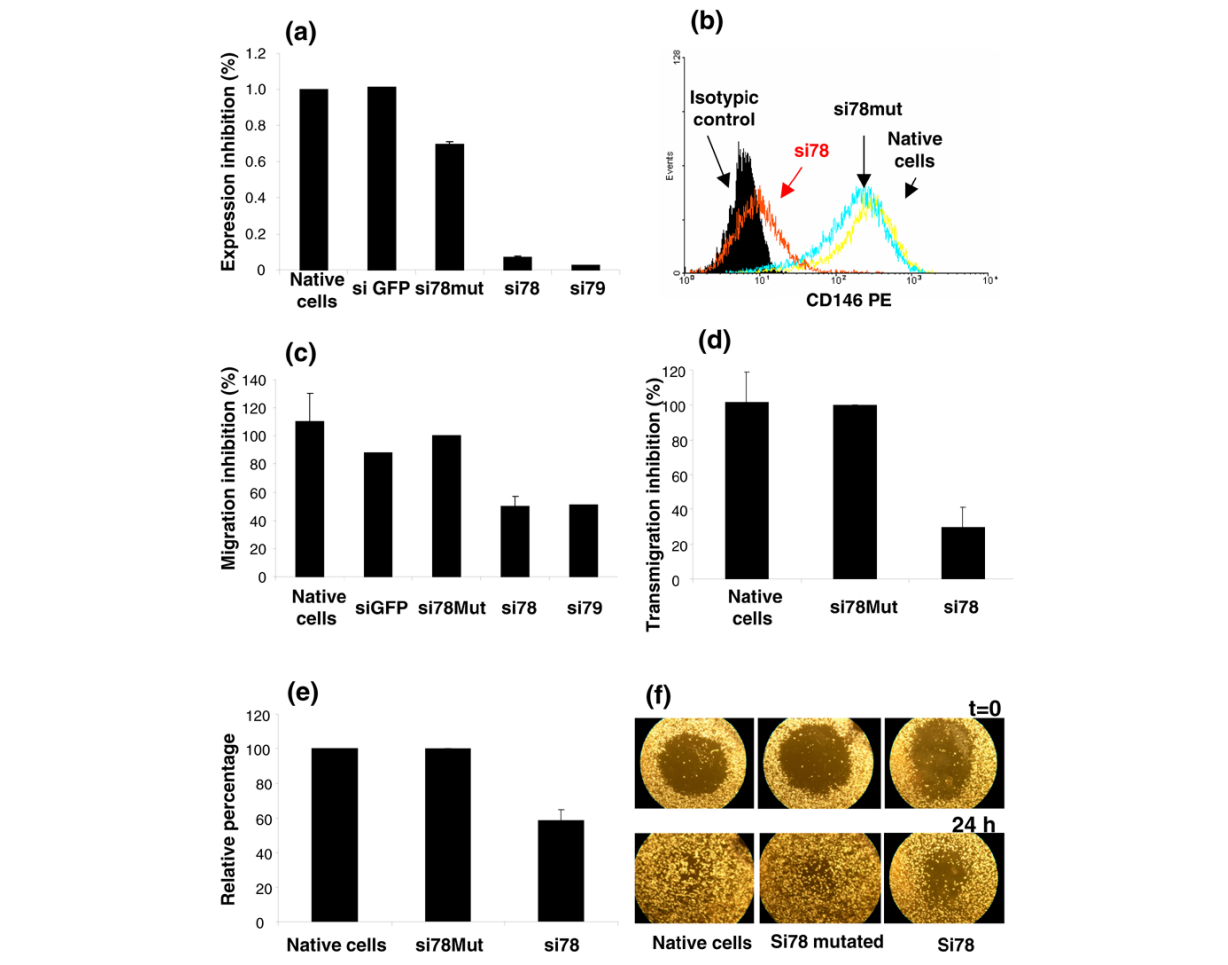
**Downmodulation of CD146 expression and migration abilities of the MDA-MB-231 cell line**. **(a) **CD146 mRNA expression in the MDA-MB-231 cell line 72 hours after transfection with siRNAs targeting CD146. Two different siRNAs (si78 and si79) and two controls (si78mut and siGFP) were used. CD146mRNA expression was normalized to GAPDH and expressed relatively to the native cells (arbitrarily 100%). **(b) **Protein expression (PE) measured by flow cytometry, one representative experiment. **(c) **Chemotactic migration evaluated after 24 hours, using uncoated Boyden chambers and 10% FCS as chemoattractant. **(d) **Transmigration through the established human endothelial HBMEC cell line. **(e) **and **(f) **Wound healing assay. (e) Wound healing repair was evaluated after 24 hours; percentage of the initial wound surface repaired after 24 hours was estimated using ImageJ software. (f) A representative experiment of wound healing: MDA-MB-231 cells were transfected with siRNAs and, 3 days after transfection, a cell-free area (wound) was created in confluent cultures (*t *= 0); cells were allowed to migrate for 24 hours (*t *= 24) before image analysis. For (c), (d) and (e) results are expressed relative to the results obtained with the mutated si78 RNA (arbitrarily 100%) and represent the mean ± standard error of the mean of four independent experiments (except for (d), which represents two experiments).

CD146 expression was downmodulated by transient transfection with siRNAs. The two most efficient siRNAs (si78 and si79) produced a significant reduction in the levels of CD146 mRNA and protein (Figure 6a,b) when compared with three control cell lines: the wild-type cell line, and two cell lines transfected with control siRNAs that respectively target green fluorescent protein or differ from si78 by four nucleotides in its sequence (si78mut).

The downregulation of CD146 expression was associated with reduced abilities to migrate in a transwell assay in response to 10% FCS when compared with wild-type cells or mock-transfected cells (Figure 6c). In addition, exposure of wild-type MDA-MB-231 cells to S-endo1 anti-CD146 mAb resulted in a decreased migration (n = 4; data not shown); in one additional experiment, the S-endo1 mAb had the same effect on the Sum159PT cell line (data not shown). Downmodulation of CD146 expression also resulted in a significant decrease of MDA-MB-231 transmigration through the established HBMEC human endothelial cell line (Figure 6d). Finally, MDA-MB-231 cells also displayed a reduced ability to heal a wound, following CD146 downmodulation (Figure 6e,f).

### Downmodulation of CD146 expression results in changes in the expression signature of the MDA-MB-231 breast cancer cell line

We generated two MDA-MB-231-derived cell lines (TI6194 and TI6196) that stably expressed 29mer shRNAs against CD146. Knockdown of CD146 was confirmed at the mRNA level using quantitative RT-PCR (see Figure S1a in Additional data file 5) and whole-genome DNA microarrays (Table 2). Phenotypic analysis of CD146 expression showed that the mean fluorescence intensity of CD146 was reduced more than 80%, the inhibition being greater in the TI6194 cell line (see Figure S1b in Additional data file 5). Empty plasmid or shRNA directed against green fluorescent protein did not affect CD146 expression. Migration in a transwell assay of TI6194 and TI6196 cells decreased accordingly (data not shown).

**Table 2:** Genes modulated as a consequence of CD146 downmodulation

Symbol	Accession number	Fold change	*P *value (*t *test)
DNA microarray			
LIPG	[GenBank:NM_006033]	-6.75	0.02749
MCAM	[GenBank:NM_006500]	-3.61	0.00084
FPR1	[GenBank:NM_002029]	-2.88	0.03159
CYP1B1	[GenBank:NM_000104]	-2.73	0.03208
FN1	[GenBank:NM_212475]	-2.48	0.00657
SORCS2	[GenBank:NM_020777]	-2.17	0.0314
FLJ20160	[GenBank:NM_017694]	-1.74	0.01431
LTBP1	[GenBank:NM_206943]	-1.72	0.03606
HES1	[GenBank:NM_005524]	-1.7	0.04315
AIM1	[GenBank:NM_001624]	-1.63	0.00758
MFAP2	[GenBank:NM_002403]	-1.58	0.04437
HIPK2	[GenBank:NM_022740]	-1.55	0.00781
CALD1	[GenBank:NM_033138]	-1.51	0.0113
NAV1	[GenBank:NM_020443]	-1.5	0.00481
NFIC	[GenBank:NM_005597]	-1.5	0.0275
TGFBI	[GenBank:NM_000660]	-1.85*	0.03459
TRIM59	[GenBank:NM_173084]	-1.76*	0.01626
NFAT5	[GenBank:NM_138714]	-1.51*	0.04496
APCDD1L	[GenBank:NM_153360]	-2.38**	0.01035
GARNL4	[GenBank:NM_001100398]	-1.96**	0.04209
PCMTD1	[GenBank:NM_052937]	-1.59**	0.0362
Multiplex quantitative RT-PCR			
KALRN	[GenBank:NM_003947]	-10	0.02973
PREX1	[GenBank:NM_020820]	-8.93	0.01711
VAV1	[GenBank:NM_005428]	-7.94	0.00688
FGD4	[GenBank:NM_139241]	-4.63	0.00318
RHOV	[GenBank:NM_133639]	-4.03	0.03116
CCL5	[GenBank:NM_002985]	-3.88	0.00536
ITGB3	[GenBank:NM_000212]	-3.61	0.00187
CXCL16	[GenBank:NM_022059]	-2.92	0.00252
THBS1	[GenBank:NM_003246]	-2.66	0.01279
CXCR4	[GenBank:NM_001008540]	-2.21	0.00259
ITGB4	[GenBank:NM_001005731]	-2.15	0.01068
CD44	[GenBank:NM_000610]	-1.92	0.00141
ITGA2	[GenBank:NM_002203]	-1.86	0.00191
ROCK2	[GenBank:NM_004850]	-1,80	0.00004
ITGA6	[GenBank:NM_000210]	-1.63	0.00274
VIM	[GenBank:NM_003380]	-1.45***	0.04761
ARHGEF11	[GenBank:NM_198236]	1.67	0.00288
BCL7A	[GenBank:NM_001024808]	1.69	0.02473
DEF6	[GenBank:NM_022047]	2.05	0.00946
TNK2	[GenBank:NM_005781]	2.18	0.00222
CXCL10	[GenBank:NM_001565]	2.51	0.03645

*Not validated by quantitative RT-PCR. **Quantitative RT-PCR not available.***Observed only with the TI6194 cell line.

Wild-type, mock-transfected and TI6194 and TI6196 transfected cells were profiled using whole-genome DNA microarrays to identify genes whose expression was modified by CD146 downregulation. The results are shown in a volcano plot (see Additional data file 6). Supervised analysis using the *t *test revealed 363 probe sets differentially expressed (*P *< 0.05 with false discovery rate correction). Among these, 13 probe sets (Table 2) displayed a ≥ 2-fold decrease associated with CD146 downregulation. These 13 probe sets represented seven unique genes including MCAM/CD146. With a less stringent cutoff value (≥ 1.5-fold change), 28 probe sets – representing 21 unique genes – were downregulated in the cell lines with CD146 inhibition (Table 2). Strikingly, no probe set was upregulated in these cell lines. Quantitative RT-PCR confirmed these results for 15 out of the 21 tested genes, failed for three genes, and did not identify any significant differential expression for three genes (Table 2).

To further explore mRNA expression changes related to the downmodulation in CD146, we used multiplex quantitative RT-PCR. We focused the analysis on the expression of 184 genes involved in migration, adhesion and cytoskeleton. The genes whose expression was significantly modulated in this screening belong to different protein families, including integrins (α2, α6, β3, β4 subunits), extracellular matrix or structural proteins, transcription factors, and molecules involved in signal transduction (Table 2). The expression of Vimentin, a mesenchymal marker (like fibronectin 1), was downregulated in the TI6194 cell line, which is the engineered MDA-MB-231 cell line with the lowest expression of CD146. Interestingly, the downregulation of CD146 expression was associated with a downregulation of the proto-oncogene VAV1, of CXCR4 (the receptor for CXCL12) and of the chemokine CCL5. Only few transcripts were upregulated: among these transcripts, DEF6, CXCL10 and BCL7A are associated with a lower tumorigenicity.

There was a good correlation between the results obtained with the two expression profiling approaches. Out of the 99 genes represented on multiplex plates and Affymetrix microarrays (among the 18,041 probe sets retained after the filtering step), 85 genes (86%) displayed concordant results according to the two approaches – whereas only 14 genes showed discordant results. This discordance may be related to the difference in sensitivity of the two techniques, as well as to different transcripts tested. The downregulation of transcripts for CXCR4, CD44, ICAM1 and integrin α6 was confirmed by cytometry analyses, and the upregulation of CXCL10 was confirmed using an antibody array (data not shown).

## Discussion

MCAM or CD146, a cell–cell or cell–matrix adhesion molecule, was first described in melanomas where a high level of CD146 expression is associated with a poor prognosis [35]. More recently, the high expression of CD146 has also been associated with metastatic progression in prostate cancer [14] and ovarian cancer [16]. A previous report supports a tumor suppressor role rather than a prometastatic role for CD146 in breast cancer pathogenesis [23].

Tissue microarray analysis indicates that only a small subset of human primary tumors expresses CD146 proteins in the epithelial compartment. CD146 expression correlates with a high tumor grade and triple-negative receptor status. A correlation was also observed with epithelial growth factor receptor, P-cadherin, p53, Moesin, Bcl2 and GATA3 expression. All of these characteristics are associated with the basal phenotype. In the present study of 635 breast tumors, CD146 expression is associated with a poor overall survival with a follow-up cutoff point at 5 years.

As revealed by DNA microarrays, CD146 belongs to a stromal/mesenchymal signature and is expressed in human mammary tumor cell lines that can be classified in the basal-B subtype [36]. Then, similarly to other tumor types, CD146 expression is associated with poor prognosis in breast cancers. A possible explanation for this association is the role of CD146 in cell motility. First, the correlation between CD146 expression and the presence of CD44^+^/CD24^low ^cells in breast cancer cell lines (data not shown) is consistent with previous observations at the mRNA level in cell lines [36], and supports the hypothesis that CD146 expression may be associated with the metastatic potential of breast tumor cells [37].

Downmodulation of CD146 expression by siRNA or shRNA approaches in cell lines that spontaneously express a high level of CD146 mostly resulted in a decreased migration in three distinct assays, at least one of which involves heterotypic cell–cell interactions. These results in breast cancer are consistent with previously reported results for melanoma cells [38,39]. Although the exact function of CD146 on different cell types remains elusive, these observations – together with published results for lymphocytes [8] – reinforce the hypothesis that CD146 is an actor of cell migration. Witze and colleagues recently showed that CD146 is recruited with actin, myosin IIB and Frizzled into an intracellular structure, which accumulates at the cell periphery and is associated with membrane retraction in response to Wnt5a [39].

Changes in molecular signatures following downmodulation of CD146 expression support the contribution of indirect mechanisms in decreased migration abilities. A small subset of genes appeared to be mostly downmodulated. Some of these genes are obvious players in cell migration, including CD44 [40] and FPR1 [41]. Concomitantly with CD146 downmodulation, we observed a downmodulation of CXCR4 and CCL5. CXCR4 is known to be upregulated in breast tumors; neutralizing the interaction of CXCL12 with CXCR4 significantly reduces breast cancer cell metastases *in vivo *[42], as does the repression of CCL5 that has anti-migratory effects [43]. Overexpression of CCL5 in MDA-MB-231 cells enhances their metastatic potential [44].

Other modulated genes are involved in oncogenic processes: CYP1B1 is associated with adverse prognosis [45], and VAV1 is a known proto-oncogene. Among the few upregulated genes detected with quantitative RT-PCR, BCL7A has been classified as a tumor suppressor gene [46], while CXCL10 mediates a thymus-dependent antitumor response *in vivo *[47].

Downmodulation of CD146 induces the downmodulation of genes such as vimentin, fibronectin and thrombospondin, which are mesenchymal markers; it also induces downmodulation of LTBP1, a protein expressed in stromal cells that triggers the biological activity of tumor growth factor beta. Altogether, these observations suggest that CD146 downmodulation is associated with the reversal of several biological characteristics associated with an aggressive phenotype.

## Conclusion

We provide evidence that CD146 is involved in breast cancer cell line motility and is associated with the basal subtype of primary breast cancers. In this picture, CD146 appears as a prometastatic factor associated with poor-prognosis histoclinical features, rather than as a tumor suppressor gene. Because CD146 downmodulation is associated with the reversal of several biological characteristics leading to a less aggressive phenotype, treatments targeting CD146 could be considered in breast cancers as in other malignancies.

## Abbreviations

ER: estrogen receptor; FCS: fetal calf serum; mAb: monoclonal antibody; PR: progesterone receptor; RT-PCR: real-time polymerase chain reaction; RPMI: Roswell Park Memorial Institute; shRNA: short hairpin RNA; siRNA: small inhibitory RNA.

## Competing interests

The authors declare that they have no competing interests.

## Authors' contributions

GZ and AM-I contributed equally. GZ and AM-I designed and performed *in vitro *experiments, and wrote the manuscript. PF and FB performed and interpreted DNA microarrays experiments. JJ performed and interpreted tissue microarray experiments. TM helped in the validation of siRNA. BE performed statistical analyses. DB reviewed and discussed the results. CC designed experiments, reviewed results, and wrote and edited the manuscript.

## Authors' information

The present work was presented in part at the 2007 Annual Meeting of the American Association for Cancer Research in Los Angeles, USA (abstract # 5711).

## Supplementary Material

Additional file 1 A Word file containing information about the mAbs used in the present study. Click here for file

Additional file 2 A Word file containing information about the sequences of primers used in quantitative RT-PCR and sequences of siRNAs and shRNAs. Click here for file

Additional file 3 A Word file containing a table that presents the genes tested by quantitative RT-PCR. Click here for file

Additional file 4 A Word file containing a table that presents the genes of the stromal cluster. Click here for file

Additional file 5 An Adobe file containing a figure showing the downmodulation of CD146 in MDA-MB-231 cells with shRNAs targeting CD146. Click here for file

Additional file 6 An Adobe file containing a figure showing the mRNA expression levels between the wild-type and the mock-transfected MDA-MB-231 cell lines displayed as a volcano plot. Click here for file
