# Supplementary material for: CD146 expression is associated with a poor prognosis in human breast tumors and with enhanced motility in breast cancer cell lines
Source: Breast Cancer Res. 2009 Jan 5;11(1):R1. doi: 10.1186/bcr2215 (PMC2687703; doi:10.1186/bcr2215)
Supplement: Additional file 1 — A Word file containing information about the mAbs used in the present study. [file bcr2215-S1.doc]

**Additional data file 1:** antibodies used in this study

| **Antibodies** | **Clone** |  | **Supplier** |  |
| --- | --- | --- | --- | --- |
| CD146 | P1H12 | PE | Becton-Dickinson (San Jose, CA, USA) | FC |
|  | S-Endo1 |  | BioCytex (Marseille, France) | FC |
|  | N1238 |  | Novocastra Laboratories (Newcastle, UK) | TMA |
| CD24 | ALB9 | PE | Beckman-Coulter (Miami, FL, USA) | FC |
| CD44 | J173 | PE | Beckman-Coulter | FC |
| CD49f (integrin α6) | GoH3 | PE | Becton-Dickinson | FC |
| CD54 (ICAM-1) | 84H10 | FITC | Beckman-Coulter | FC |
| CXCR4 | 12G5 | PE | R&D Systems (Minneapolis, MN, USA) | FC |
| Bcl2 | 124 |  | Dako (Dako France S.A.S., Trappes, France) |  |
| EGFR | 31G7 |  | Invitrogen (Paisley, UK) | TMA |
| ERBB2 | CB11 |  | Novocastra Laboratories | TMA |
| Estrogen receptor (ER) | 6F11 |  | Novocastra Laboratories | TMA |
| Gata3 | sc-268 |  | Santa Cruz Biotechnology (Santa Cruz, CA, USA) | TMA |
| Moesin | 38/87 |  | Biomeda (Foster City, CA, USA) | TMA |
| P53 | DO1 |  | Dako | TMA |
| P-Cadherin | 56 |  | Becton-Dickinson | TMA |
| Progesterone receptor (PR) | PgR636 |  | Dako | TMA |
| FC = flow cytometry, TMA = tissue microarray | | | | |
