# Supplementary material for: CD146 expression is associated with a poor prognosis in human breast tumors and with enhanced motility in breast cancer cell lines
Source: Breast Cancer Res. 2009 Jan 5;11(1):R1. doi: 10.1186/bcr2215 (PMC2687703; doi:10.1186/bcr2215)
Supplement: Additional file 2 — A Word file containing information about the sequences of primers used in quantitative RT-PCR and sequences of siRNAs and shRNAs. [file bcr2215-S2.doc]

| Supplementary table 2 : Primers, siRNA and shRNA used in this study | | | | | | | | | |  |
| --- | --- | --- | --- | --- | --- | --- | --- | --- | --- | --- |
| cDNA | GenBank accession number | Amplicon Size |  | Primer Sequences | | | | | |  |
| AIM1 | NM_001624 | 187 | (F) | 5'-TCCTTGTCTGTCTGCAATGG-3' | | | | | |  |
|  |  |  | (R) | 5'-TGCCACCAATAACCTGAACA-3' | | | | | |  |
| CALD1 | NM_033138 | 247 | (F) | 5'-TGCAGAAAAGCAGTGGTGTC-3' | | | | | |  |
|  |  |  | (R) | 5'-TTCAAGCCAGCAGTTTCCTT-3' | | | | | |  |
| CYP1B1 | NM_000104 | 245 | (F) | 5'-TGATGGACGCCTTTATCCTC-3' | | | | | |  |
|  |  |  | (R) | 5'-CCACGACCTGATCCAATTCT-3' | | | | | |  |
| FLJ20160 | NM_017694 | 243 | (F) | 5'-TTGGTTCATGGGTTTTGGAT-3' | | | | | |  |
|  |  |  | (R) | 5'-GTCCAGGCATTCTCCAGGTA-3' | | | | | |  |
| FN1 | NM_212475 | 230 | (F) | 5'-ACCAACCTACGGATGACTCG-3' | | | | | |  |
|  |  |  | (R) | 5'-GCTCATCATCTGGCCATTTT-3' | | | | | |  |
| FPR1 | NM_002029 | 177 | (F) | 5'-CTGAGTCACTCTCCCCAGGA-3' | | | | | |  |
|  |  |  | (R) | 5'-CCAGGAAGAGATAGCCAGCA-3' | | | | | |  |
| HES1 | NM_005524 | 222 | (F) | 5'-CGGACATTCTGGAAATGACA-3' | | | | | |  |
|  |  |  | (R) | 5'-CATTGATCTGGGTCATGCAG-3' | | | | | |  |
| HIPK2 | NM_022740 | 212 | (F) | 5'-CGGGAGTTCATTGACCTGTT-3' | | | | | |  |
|  |  |  | (R) | 5'-AAAGGGGTTTTGCTCTGGTT-3' | | | | | |  |
| LIPG | NM_006033 | 150 | (F) | 5'-TCAACGATGTCTTGGGATCA-3' | | | | | |  |
|  |  |  | (R) | 5'-TGAAGCGATTGGAGTCAGTG-3' | | | | | |  |
| LTBP1 | NM_206943 | 220 | (F) | 5'-AATACCATGGGCAGCTATCG-3' | | | | | |  |
|  |  |  | (R) | 5'-CTGGAAGGGGACATTTCTCA-3' | | | | | |  |
| MCAM | NM_006500 | 222 | (F) | 5'-GGGTACCCCATTCCTCAAGT-3' | | | | | |  |
|  |  |  | (R) | 5'-CCTGGACTCCTTCATGTGGT-3' | | | | | |  |
| MFAP2 | NM_002403 | 223 | (F) | 5'-CCAGATCGACAACCCAGACT-3' | | | | | |  |
|  |  |  | (R) | 5'-GCAAGGCCTGTGTATGGAGT-3' | | | | | |  |
| NAV1 | NM_020443 | 238 | (F) | 5'-TCAAGCCTCAACAGCATCAC-3' | | | | | |  |
|  |  |  | (R) | 5'-TGGAGGGTGAAGCAGTCTCT-3' | | | | | |  |
| NFAT5 | NM_138714 | 161 | (F) | 5'-'TGAGGGAAAGGAGCTGAAGA-3' | | | | | |  |
|  |  |  | (R) | 5'-TCACTCGTCCAGAGTCGTTG-3' | | | | | |  |
| NFIC | NM_005597 | 250 | (F) | 5'-ACCTGGCATACGACCTGAAC-3' | | | | | |  |
|  |  |  | (R) | 5'-GGGCTGTTGAATGGTGACTT-3' | | | | | |  |
| SORCS2 | NM_020777 | 238 | (F) | 5'-CATTCCCTTCTTCGTGGAAA-3' | | | | | |  |
|  |  |  | (R) | 5'-GCAGGTGACGTACCGAAAAT-3' | | | | | |  |
| TGFB1 | NM_000660 | 156 | (F) | 5'-GGGACTATCCACCTGCAAGA-3' | | | | | |  |
|  |  |  | (R) | 5'-CCTCCTTGGCGTAGTAGTCG-3' | | | | | |  |
| TRIM59 | NM_173084 | 234 | (F) | 5'-TCCCCGCGAATAAGAATAAA-3' | | | | | |  |
|  |  |  | (R) | 5'-GAGAGCATGGCAGTACACGA-3' | | | | | |  |
| siRNA against MCAM (GenBank NM_006500] | | | | |  |  | |  | |  |
| si78 |  |  |  | 5'-UUCAACACCAUAUUCUCUUUCACCC-3' | | | | | |  |
| si79 |  |  |  | 5'-AAACCAGUCGACAUGGCUGAGGUUG-3' | | | | | |  |
| si78mut |  |  |  | 5'-UUCAACACCAU**UACU**UCUUUCACCC-3' | | | | | |  |
| siGFP |  |  |  | 5'-GCAAGCUGACCCUGAAGUUCAU-3' | | | | | |  |
| shRNA against MCAM (GenBank NM_006500] | | | | |  |  | |  | |  |
| TI6194 |  |  |  | CACCTTGCAGAGTATTCTGAAGGCACAGC | | | | | |  |
| TI6196 |  |  |  | GGAGAGAAATACATCGATCTGAGGCATTA | | | | | |  |
| Bold characters indicate the four modified nucleotides compared to si78. | | | | | | |  | |  | |
