# Supplementary material for: CD146 expression is associated with a poor prognosis in human breast tumors and with enhanced motility in breast cancer cell lines
Source: Breast Cancer Res. 2009 Jan 5;11(1):R1. doi: 10.1186/bcr2215 (PMC2687703; doi:10.1186/bcr2215)
Supplement: Additional file 3 — A Word file containing a table that presents the genes tested by quantitative RT-PCR. [file bcr2215-S3.doc]

**Additional data file 3:** List of Q-RT-PCR tested genes.

| **symbole** | **Genbank accession number** | **description** |
| --- | --- | --- |
| *ABRA* | NM_139166 | actin-binding Rho activating protein |
| *AKAP13* | NM_007200 | A kinase (PRKA) anchor protein 13 |
| *ARHGEF1* | NM_006421 | Rho guanine nucleotide exchange factor (GEF) 1 |
| *ARHGEF11* | NM_198236 | Rho guanine nucleotide exchange factor (GEF) 11 |
| *ARHGEF12* | NM_015313 | Rho guanine nucleotide exchange factor (GEF) 12 |
| *ARHGEF2* | NM_004723 | rho/rac guanine nucleotide exchange factor (GEF) 2 |
| *ARHGEF4* | NM_015320 | Rho guanine nucleotide exchange factor (GEF) 4 |
| *ARHGEF7* | NM_001113511 | Rho guanine nucleotide exchange factor (GEF) 7 |
| *BCL7A* | NM_001024808 | B-cell CLL/lymphoma 7A |
| *BCR* | NM_004237 | breakpoint cluster region |
| *CCL1* | NM_002981 | chemokine (C-C motif) ligand 1 |
| *CCL11* | NM_002986 | chemokine (C-C motif) ligand 11 |
| *CCL17* | NM_002987 | chemokine (C-C motif) ligand 17 |
| *CCL19* | NM_006274 | chemokine (C-C motif) ligand 19 |
| *CCL2* | NM_002982 | chemokine (C-C motif) ligand 2 |
| *CCL20* | NM_004591 | chemokine (C-C motif) ligand 20 |
| *CCL21* | NM_002989 | chemokine (C-C motif) ligand 21 |
| *CCL22* | NM_002990 | chemokine (C-C motif) ligand 22 |
| *CCL24* | NM_002991 | chemokine (C-C motif) ligand 24 |
| *CCL25* | NM_005624 | chemokine (C-C motif) ligand 25 |
| *CCL27* | NM_006664 | chemokine (C-C motif) ligand 27 |
| *CCL28* | NM_148672 | chemokine (C-C motif) ligand 28 |
| *CCL3* | NM_002983 | chemokine (C-C motif) ligand 3 |
| *CCL4* | NM_002984 | chemokine (C-C motif) ligand 4 |
| *CCL5* | NM_002985 | chemokine (C-C motif) ligand 5 |
| *CCL7* | NM_006273 | chemokine (C-C motif) ligand 7 |
| *CCL8* | NM_005623 | chemokine (C-C motif) ligand 8 |
| *CCR1* | NM_001295 | chemokine (C-C motif) receptor 1 |
| *CCR10* | NM_016602 | chemokine (C-C motif) receptor 10 |
| *CCR2* | NM_000647 | chemokine (C-C motif) receptor 2 |
| *CCR3* | NM_001837 | chemokine (C-C motif) receptor 3 |
| *CCR4* | NM_005508 | chemokine (C-C motif) receptor 4 |
| *CCR5* | NM_000579 | chemokine (C-C motif) receptor 5 |
| *CCR6* | NM_004367 | chemokine (C-C motif) receptor 6 |
| *CCR7* | NM_001838 | chemokine (C-C motif) receptor 7 |
| *CCR8* | NM_005201 | chemokine (C-C motif) receptor 8 |
| *CCR9* | NM_006641 | chemokine (C-C motif) receptor 9 |
| *CCRL1* | NM_016557 | chemokine (C-C motif) receptor-like 1 |
| *CD151* | NM_004357 | CD151 molecule (Raph blood group) |
| *CD37* | NM_001040031 | CD37 molecule |
| *CD44* | NM_000610 | CD44 molecule (Indian blood group) |
| *CD47* | NM_001777 | CD47 molecule |
| *CD63* | NM_001040034 | CD63 molecule |
| *CD81* | NM_004356 | 11pter-p11.2 |
| *CD9* | NM_001769 | CD9 molecule |
| *CDC42* | NM_001791 | cell division cycle 42 (GTP binding protein, 25kDa) |
| *CX3CL1* | NM_002996 | chemokine (C-X3-C motif) ligand 1 |
| *CX3CR1* | NM_001337 | chemokine (C-X3-C motif) receptor 1 |
| *CXCL1* | NM_001511 | chemokine (C-X-C motif) ligand 1 (melanoma growth stimulating activity, alpha) |
| *CXCL10* | NM_001565 | chemokine (C-X-C motif) ligand 10 |
| *CXCL11* | NM_005409 | chemokine (C-X-C motif) ligand 11 |
| *CXCL12* | NM_000609 | chemokine (C-X-C motif) ligand 12 (stromal cell-derived factor 1) |
| *CXCL13* | NM_006419 | chemokine (C-X-C motif) ligand 13 (B-cell chemoattractant) |
| *CXCL14* | NM_004887 | chemokine (C-X-C motif) ligand 14 |
| *CXCL16* | NM_022059 | chemokine (C-X-C motif) ligand 16 |
| *CXCL5* | NM_002994 | chemokine (C-X-C motif) ligand 5 |
| *CXCL9* | NM_002416 | chemokine (C-X-C motif) ligand 9 |
| *CXCR3* | NM_001504 | chemokine (C-X-C motif) receptor 3 |
| *CXCR4* | NM_0010085400 | chemokine (C-X-C motif) receptor 4 |
| *CXCR5* | NM_001716 | chemokine (C-X-C motif) receptor 5 |
| *CXCR6* | NM_006564 | chemokine (C-X-C motif) receptor 6 |
| *DARC* | NM_002036 | Duffy blood group, chemokine receptor |
| *DBNL* | NM_014063 | drebrin-like |
| *DEF6* | NM_022047 | differentially expressed in FDCP 6 homolog (mouse) |
| *DEPDC2* | NM_025170 | DEP domain containing 2 |
| *DGKD* | NM_003648 | diacylglycerol kinase, delta 130kDa |
| *DIAPH1* | NM_005219 | diaphanous homolog 1 (Drosophila) |
| *DIAPH2* | NM_007309 | diaphanous homolog 2 (Drosophila) |
| *DNMBP* | NM_015221 | dynamin binding protein |
| *DOCK2* | NM_004946 | dedicator of cytokinesis 2 |
| *DOCK9* | NM_015296 | dedicator of cytokinesis 9 |
| *ECT2* | NM_018098 | epithelial cell transforming sequence 2 oncogene |
| *EZR* | NM_003379 | ezrin |
| *FARP2* | NM_014808 | FERM, RhoGEF and pleckstrin domain protein 2 |
| *FGD1* | NM_004463 | FYVE, RhoGEF and PH domain containing 1 |
| *FGD3* | NM_033086 | FYVE, RhoGEF and PH domain containing 3 |
| *FGD4* | NM_139241 | FYVE, RhoGEF and PH domain containing 4 |
| *FSCN2* | NM_012418 | fascin homolog 2, actin-bundling protein, retinal (Strongylocentrotus purpuratus) |
| *GPR44* | NM_004778 | G protein-coupled receptor 44 |
| *IL8* | NM_000584 | interleukin 8 |
| *IL8RA* | NM_000634 | interleukin 8 receptor, alpha |
| *IL8RB* | NM_001557 | interleukin 8 receptor, beta |
| *IQGAP1* | NM_003870 | IQ motif containing GTPase activating protein 1 |
| *ITGA1* | NM_181501 | integrin, alpha 1 |
| *ITGA2* | NM_002203 | integrin, alpha 2 (CD49B, alpha 2 subunit of VLA-2 receptor) |
| *ITGA2B* | NM_000419 | integrin, alpha 2b (platelet glycoprotein IIb of IIb/IIIa complex, antigen CD41) |
| *ITGA3* | NM_005501 | integrin, alpha 3 (antigen CD49C, alpha 3 subunit of VLA-3 receptor) |
| *ITGA4* | NM_000885 | integrin, alpha 4 (antigen CD49D, alpha 4 subunit of VLA-4 receptor) |
| *ITGA5* | NM_002205 | integrin, alpha 5 (fibronectin receptor, alpha polypeptide) |
| *ITGA6* | NM_000210 | integrin, alpha 6 |
| *ITGA7* | NM_002206 | integrin, alpha 7 |
| *ITGA8* | NM_003638 | integrin, alpha 8 |
| *ITGAE* | NM_002208 | integrin, alpha E (antigen CD103, human mucosal lymphocyte antigen 1; alpha polypeptide) |
| *ITGAL* | NM_002209 | integrin, alpha L (antigen CD11A (p180), lymphocyte function-associated antigen 1; alpha polypeptide) |
| *ITGAM* | NM_000632 | integrin, alpha M (complement component 3 receptor 3 subunit) |
| *ITGAV* | NM_002210 | integrin, alpha V (vitronectin receptor, alpha polypeptide, antigen CD51) |
| *ITGAX* | NM_000887 | integrin, alpha X (complement component 3 receptor 4 subunit) |
| *ITGB1* | NM_002211 | integrin, beta 1 (fibronectin receptor, beta polypeptide, antigen CD29 includes MDF2, MSK12) |
| *ITGB2* | NM_000211 | integrin, beta 2 (complement component 3 receptor 3 and 4 subunit) |
| *ITGB3* | NM_000212 | integrin, beta 3 (platelet glycoprotein IIIa, antigen CD61) |
| *ITGB4* | NM_001005731 | integrin, beta 4 |
| *ITGB5* | NM_002213 | integrin, beta 5 |
| *ITGB6* | NM_000888 | integrin, beta 6 |
| *ITGB7* | NM_000889 | integrin, beta 7 |
| *ITGB8* | NM_002214 | integrin, beta 8 |
| *ITSN1* | NM_003024 | intersectin 1 (SH3 domain protein) |
| *ITSN2* | NM_006217 | intersectin 2, SWAP 70 |
| *KALRN* | NM_003947 | kalirin, RhoGEF kinase |
| *LIMK1* | NM_002314 | LIM domain kinase 1 |
| *LTB4R* | NM_181657 | leukotriene B4 receptor |
| *MAP3K1* | NM_042066 | mitogen-activated protein kinase kinase kinase 1 |
| *MAP3K10* | NM_002446 | mitogen-activated protein kinase kinase kinase 10 |
| *MAP3K11* | NM_002419 | mitogen-activated protein kinase kinase kinase 11 |
| *MCAM* | NM_006500 | melanoma cell adhesion molecule |
| *MCF2* | NM_005369 | MCF.2 cell line derived transforming sequence |
| *MIF* | NM_002415 | macrophage migration inhibitory factor (glycosylation-inhibiting factor) |
| *MSN* | NM_002444 | moesin |
| *NCK1* | NM_006153 | NCK adaptor protein 1 |
| *NCK2* | NM_003581 | NCK adaptor protein 2 |
| *NET1* | NM_005863 | neuroepithelial cell transforming gene 1 |
| *PAK1* | NM_002576 | p21 protein (Cdc42/Rac)-activated kinase 1 |
| *PAK2* | NM_002577 | p21 protein (Cdc42/Rac)-activated kinase 2 |
| *PAK3* | NM_002578 | p21 protein (Cdc42/Rac)-activated kinase 3 |
| *PAK4* | NM_001014831 | p21 protein (Cdc42/Rac)-activated kinase 4 |
| *PARD6A* | NM_016948 | par-6 partitioning defective 6 homolog alpha (C. elegans) |
| *PECAM1* | NM_000442 | platelet/endothelial cell adhesion molecule |
| *PF4* | NM_002619 | platelet factor 4 (chemokine (C-X-C motif) ligand 4) |
| *PKN1* | NM_002741 | protein kinase N1 |
| *PLCB2* | NM_004573 | phospholipase C, beta 2 |
| *PPP1R12A* | NM_002480 | protein phosphatase 1, regulatory (inhibitor) subunit 12A |
| *PREX 1* | NM_020820 | phosphatidylinositol 3,4,5-triphosphate-dependent RAC exchanger 1 |
| *RAC1* | NM_018890 | ras-related C3 botulinum toxin substrate 1 (rho family, small GTP binding protein Rac1) |
| *RAC2* | NM_002872 | ras-related C3 botulinum toxin substrate 2 (rho family, small GTP binding protein Rac2) |
| *RAC3* | NM_005052 | ras-related C3 botulinum toxin substrate 3 (rho family, small GTP binding protein Rac3) |
| *RASGRF2* | NM_006909 | Ras protein-specific guanine nucleotide-releasing factor 2 |
| *RHOA* | NM_001664 | ras homolog gene family, member A |
| *RHOB* | NM_004040 | ras homolog gene family, member B |
| *RHOBTB1* | NM_001032380 | Rho-related BTB domain containing 1 |
| *RHOBTB2* | NM_015178 | Rho-related BTB domain containing 2 |
| *RHOC* | NM_175744 | ras homolog gene family, member C |
| *RHOD* | NM_014578 | ras homolog gene family, member D |
| *RHOF* | NM_019034 | ras homolog gene family, member F (in filopodia) |
| *RHOG* | NM_001665 | ras homolog gene family, member G (rho G) |
| *RHOH* | NM_004310 | ras homolog gene family, member H |
| *RHOJ* | NM_020663 | ras homolog gene family, member J |
| *RHOQ* | NM_012249 | ras homolog gene family, member Q |
| *RHOT1* | NM_018307 | ras homolog gene family, member T1 |
| *RHOT2* | NM_138769 | ras homolog gene family, member T2 |
| *RHOU* | NM_021205 | ras homolog gene family, member U |
| *RHOV* | NM_133639 | ras homolog gene family, member U |
| *RHPN2* | NM_033103 | rhophilin, Rho GTPase binding protein 2 |
| *RIPK2* | NM_003821 | receptor-interacting serine-threonine kinase 2 |
| *RND1* | NM_014470 | Rho family GTPase 1 |
| *RND2* | NM_005440 | Rho family GTPase 2 |
| *RND3* | NM_005168 | Rho family GTPase 3 |
| *ROCK1* | NM_005406 | Rho-associated, coiled-coil containing protein kinase 1 |
| *ROCK2* | NM_004850 | Rho-associated, coiled-coil containing protein kinase 2 |
| *RPS6KB1* | NM_003161 | ribosomal protein S6 kinase, 70kDa, polypeptide 1 |
| *RRAGD* | NM_021244 | Ras-related GTP binding D |
| *RTKN* | NM_001015055 | rhotekin |
| *SELL* | NM_000655 | selectin L |
| *SELP* | NM_003005 | selectin P (granule membrane protein 140kDa, antigen CD62) |
| *SELPLG* | NM_003006 | selectin P ligand |
| *SLIT3* | NM_003062 | slit homolog 3 (Drosophila) |
| *SNX24* | NM_014035 | sorting nexin 24 |
| *SOS1* | NM_005633 | son of sevenless homolog 1 (Drosophila) |
| *SOS2* | NM_006939 | son of sevenless homolog 2 (Drosophila) |
| *SYNJ2* | NM_003898 | synaptojanin 2 |
| *THBS1* | NM_003246 | thrombospondin 1 |
| *TIAM1* | NM_003253 | T-cell lymphoma invasion and metastasis 1 |
| *TIAM2* | NM_012454 | T-cell lymphoma invasion and metastasis 2 |
| *TNK2* | NM_005781 | tyrosine kinase, non-receptor, 2 |
| *TRIO* | NM_007118 | triple functional domain (PTPRF interacting) |
| *VAV1* | NM_005428 | vav 1 guanine nucleotide exchange factor |
| *VAV2* | NM_001134398 | vav 2 guanine nucleotide exchange factor |
| *VAV3* | NM_006113 | vav 3 guanine nucleotide exchange factor |
| *WAS* | NM_000377 | Wiskott-Aldrich syndrome (eczema-thrombocytopenia) |
| *XCL1* | NM_002995 | chemokine (C motif) ligand 1 |
| *XCR1* | NM_001024644 | chemokine (C motif) receptor 1 |
| *ZYX* | NM_003461 | zyxin |
| *ACTB* | NM_001101 | actin, beta |
| *GAPDH* | NM_002046 | glyceraldehyde-3-phosphate dehydrogenase |
| *HPRT1* | NM_000194 | hypoxanthine phosphoribosyltransferase 1 |
| *USP11* | NM_004651 | ubiquitin specific peptidase 11 |
