# Supplementary material for: CD146 expression is associated with a poor prognosis in human breast tumors and with enhanced motility in breast cancer cell lines
Source: Breast Cancer Res. 2009 Jan 5;11(1):R1. doi: 10.1186/bcr2215 (PMC2687703; doi:10.1186/bcr2215)
Supplement: Additional file 4 — A Word file containing a table that presents the genes of the stromal cluster. [file bcr2215-S4.doc]

**Additional data file 4:** stromal/vascular gene cluster

| **Symbol** | **Entrez Gene ID** | | **Name** |
| --- | --- | --- | --- |
| ACTA2 | 59 | | Actin alpha 2 |
| ACTN1 | 87 | | Actinin alpha 1 |
| ADAM12 | 8038 | | ADAM metallopeptidase domain 12 |
| ADAMTS12 | 81792 | | a disintegrin like α metalloproteinase with thrombospondin type 1 motif 12 |
| ADAMTS5 | 11096 | | a disintegrin like α metalloproteinase with thrombospondin type 1 motif 5 |
| AGTRL1 | 187 | | Angiotensin receptor like 1 |
| ANGPTL1 | 9068 | | Angiopoietin like 1 |
| ANGPTL2 | 464743 | | Angiopoietin like 2 |
| ANK2 | 287 | | Ankyrin 2 |
| ANKMY2 | 57037 | | Ankyrin repeat and MYND domain containing 2 |
| CD248 | 57124 | | tumor endothelial marker 1 |
| CEECAM1 | 51148 | | cerebral endothelial cell adhesion molecule 1 |
| COL10A1 | 1300 | | collagen, type X, alpha 1 |
| COL11A1 | 1301 | | collagen, type XI, alpha 1 |
| COL12A1 | 1303 | | collagen, type XII, alpha 1 |
| COL13A1 | 1305 | | collagen, type XIII, alpha 1 |
| COL14A1 | 7373 | | collagen, type XIV, alpha 1 |
| COL15A1 | 1306 | | collagen, type XV, alpha 1 |
| COL16A1 | 1307 | | collagen, type XVI, alpha 1 |
| COL18A1 | 80781 | | collagen, type XVIII, alpha 1 |
| COL1A1 | 1277 | | collagen, type I, alpha 1 |
| COL1A2 | 1278 | | collagen, type I, alpha 2 |
| COL3A1 | 1281 | | collagen, type III, alpha 1 |
| COL4A1 | 1282 | | collagen, type IV, alpha 1 |
| COL4A2 | 1284 | | collagen, type IV, alpha 2 |
| COL5A1 | 1289 | | collagen, type V, alpha 1 |
| COL5A2 | 1290 | | collagen, type V, alpha 2 |
| COL5A3 | 50509 | | collagen, type V, alpha 3 |
| COL6A1 | 1291 | | collagen, type VI, alpha 1 |
| COL6A2 | 1292 | | collagen, type VI, alpha 2 |
| COL6A3 | 1293 | | collagen, type VI, alpha 3 |
| COL8A1 | 1295 | | collagen, type VIII, alpha 1 |
| COL8A2 | 1296 | | collagen, type VIII, alpha 2 |
| ECM2 | 1842 | | extracellular matrix protein 2 |
| EDNRA | 1909 | | endothelin receptor type A |
| EDNRB | 1910 | | endothelin receptor type B |
| EMCN | 51705 | | endomucin |
| ENG | 2022 | | endoglin |
| EPAS1 | 2034 | | endothelial PAS domain protein 1 |
| FBLN1 | 2192 | | fibulin 1 |
| FBLN2 | 2199 | | fibulin 2 |
| FBLN5 | 10516 | | fibulin 5 |
| FBN1 | 2200 | | fibrillin 1 |
| FGF1 | 2246 | | fibroblast growth factor 1 |
| FGF2 | 2247 | | fibroblast growth factor 2 |
| FGF18 | 8817 | | fibroblast growth factor 18 |
| FIGF | 2277 | | c-fis induced growth factor (VEGFD) |
| ITGA11 | 22801 | | integrin alpha 11 |
| ITGA5 | 3678 | | integrin alpha 5 |
| ITGA7 | 3679 | | integrin alpha 7 |
| ITGA8 | 8516 | | integrin alpha 8 |
| ITGA9 | 3680 | | integrin alpha 9 |
| ITGB1 | 3688 | | integrin beta 1 |
| ITGB5 | 3693 | | integrin beta 5 |
| ITGBL1 | 9358 | | integrin, beta-like 1 |
| JAM2 | 58494 | | junctional adhesion molecule 2 |
| JAM3 | 83700 | | junctional adhesion molecule 3 |
| LAMA2 | 3908 | | laminin, alpha 2 |
| LAMA4 | 3910 | | laminin, alpha 4 |
| LAMB1 | 3912 | | laminin, beta 1 |
| LAMC1 | 3915 | | laminin, gamma 1 |
| LPAR1 | 1902 | lysophosphatidic acid receptor 1 | |
| LYVE1 | 10894 | | lymphatic vessel endothelial hyaluronan receptor 1 |
| MCAM | 4162 | | melanoma cell adhesion molecule |
| MEOX1 | 4222 | | mesenchyme homeobox 1 |
| MEOX2 | 4223 | | mesenchyme homeobox 2 |
| MMP11 | 4320 | | matrix metalloproteinase 11 |
| MMP14 | 4323 | | matrix metalloproteinase 14 |
| MMP2 | 4313 | | matrix metalloproteinase 2 |
| MMP3 | 4314 | | matrix metalloproteinase 3 |
| MSN | 4478 | | moesin |
| MXRA5 | 25878 | | matrix-remodelling associated 5 |
| MXRA7 | 439921 | | matrix-remodelling associated 7 |
| MXRA8 | 54587 | | matrix-remodelling associated 8 |
| NID1 | 131390 | | nidogen 1 |
| NID2 | 22795 | | nidogen 2 |
| NRG1 | 3084 | | neuregulin 1 |
| PEAR1 | 375033 | | platelet endothelial aggregation receptor 1 |
| TIMP2 | 7077 | | TIMP metallopeptidase inhibitor 2 |
| TIMP3 | 7078 | | TIMP metallopeptidase inhibitor 3 |
| TLN1 | 7094 | | talin 1 |
| TNS1 | 7145 | | tensin 1 |
| VIM | 7431 | | vimentin |
