# Supplementary material for: CD146 expression is associated with a poor prognosis in human breast tumors and with enhanced motility in breast cancer cell lines
Source: Breast Cancer Res. 2009 Jan 5;11(1):R1. doi: 10.1186/bcr2215 (PMC2687703; doi:10.1186/bcr2215)
Supplement: Additional file 5 — An Adobe file containing a figure showing the downmodulation of CD146 in MDA-MB-231 cells with shRNAs targeting CD146. [file bcr2215-S5.pdf]

Additional data file 5

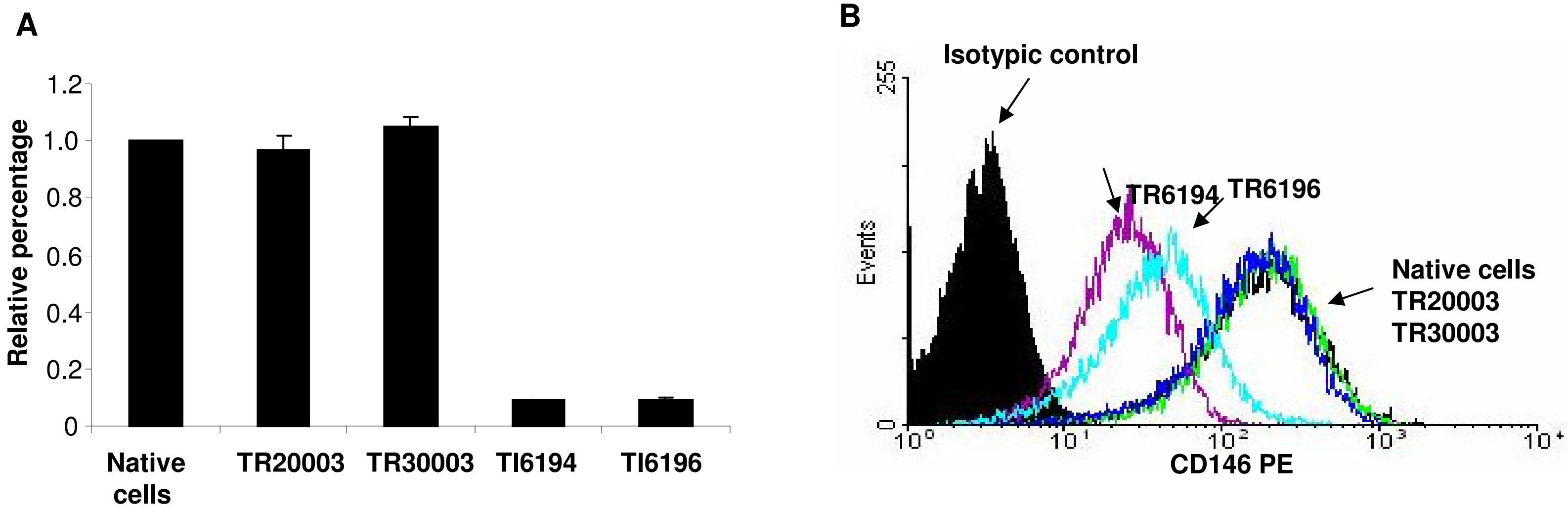

Supplementary Figure 1: Down-modulation of CD146 in MDA-MB-231 cells with shRNAs targeting CD146. Two different shRNAs (TI6194 and TI6196) and two controls (TR20003 and TR30003) were used. Cells were studied after selection with puromycin. (a) CD146 mRNA expression was measured by RQ-PCR, normalized to GAPDH and expressed relatively to the native cells (arbitrarily 100 %). (b) CD146 expression was measured by flow cytometry, one representative experiment.
