# Supplementary material for: CD146 expression is associated with a poor prognosis in human breast tumors and with enhanced motility in breast cancer cell lines
Source: Breast Cancer Res. 2009 Jan 5;11(1):R1. doi: 10.1186/bcr2215 (PMC2687703; doi:10.1186/bcr2215)
Supplement: Additional file 6 — An Adobe file containing a figure showing the mRNA expression levels between the wild-type and the mock-transfected MDA-MB-231 cell lines displayed as a volcano plot. [file bcr2215-S6.pdf]

Additional data file 6

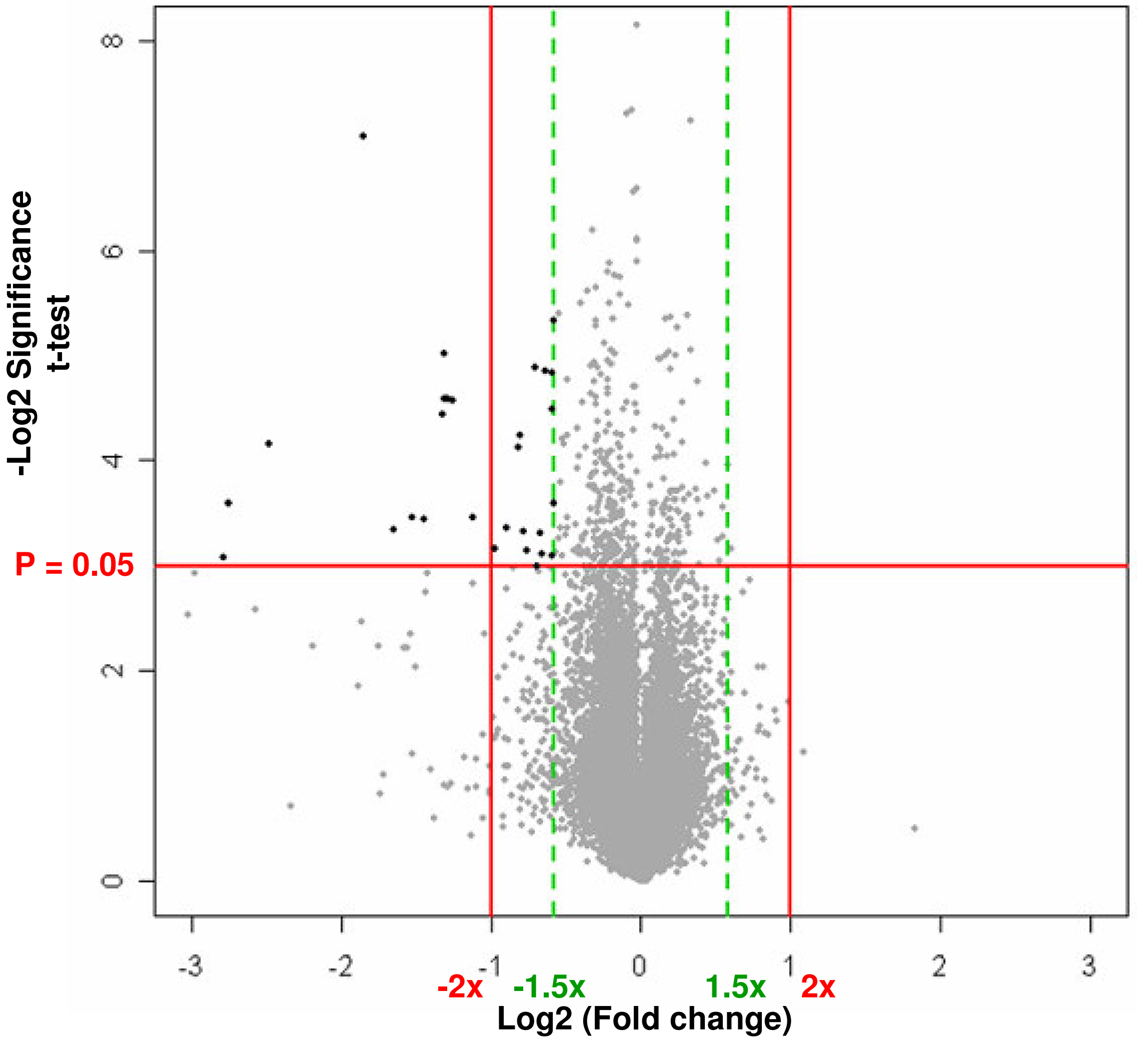

Supplementary Figure 2: Comparison of mRNA expression levels between the wild-type and the mock-transfected MDA-MB-231 cell lines displayed as a volcano plot. The volcano plot arranges the 18,041 Affymetrix probe sets (grey and black dots) retained for supervised analysis along dimensions of biological and statistical significance. The horizontal dimension (x axis) is the fold change (FC) between the two groups (on a log scale), and the vertical dimension (y axis) represents the p-value for a t-test of differences between samples (on a negative log scale so smaller p-values appear higher up). The x axis indicates biological impact of the change; the y axis indicates the statistical significance of the change. The horizontal red line indicates the retained p-value threshold (0.05 after FDR correction). The vertical colored lines represent the two FC thresholds that we used in our analysis: red for a threshold equal to 2 (see the 13 probe sets above the threshold), and green for a threshold equal to 1.5 (see the 28 probe sets above the threshold).
